# 1257. Re-Evaluation of Cefepime or Piperacillin-Tazobactam to Decrease Use of Carbapenems in ESBL-Producing Enterobacterales Urinary Tract Infections (REDUCE-UTI)

**DOI:** 10.1093/ofid/ofab466.1449

**Published:** 2021-12-04

**Authors:** Alexander C Branton, Catherine H Vu, Venugopalan Veena, Barbara A Santevecchi, Reuben Ramphal, Kartikeya Cherabuddi, Tanvi Manohar, David Rodriguez, Andrea Tello, Kathryn DeSear

**Affiliations:** 1 University of Florida Health Shands Hospital, Gainesville, Florida; 2 UF Health Shands, Belleville, New Jersey; 3 University of Florida, College of Pharmacy, Gainesville, FL; 4 University of Florida College of Pharmacy, Gainesville, Florida; 5 University of Florida College of Medicine, Gainesville, Florida; 6 University of Florida, Gainesville, FL

## Abstract

**Background:**

Carbapenems (CBP) are considered first-line for infections caused by extended-spectrum beta-lactamase-producing Enterobacterales (ESBL-E). However, recent literature suggests that cefepime (FEP) and piperacillin-tazobactam (TZP) may produce similar outcomes vs. CBPs for the treatment of ESBL-E urinary tract infections (UTIs). The goal of this study was to determine if non-carbapenem (NCBP) therapy with FEP or TZP is as effective as CBPs for the treatment of ESBL-E UTIs.

**Methods:**

This was a retrospective observational study of patients admitted to the hospital from January 1st, 2016 to June 30th, 2020 with a urine culture positive for ESBL-E. Patients were included if they received a study antibiotic (meropenem, ertapenem, TZP, or FEP). Patients were excluded if they had any of the following: absence of pyuria, prior receipt of study antibiotic, CBP-resistant organism isolated in urine culture, polymicrobial urine culture, end-stage renal disease, or concomitant gram-negative infection. The primary outcome was clinical cure defined as complete resolution of signs and symptoms of infection. Secondary outcomes included in-hospital mortality, recurrence within 30 days, and resistance within 30 days.

**Results:**

A total of 133 patients were included based on definitive therapy received; 69 (52%) received CBP and 64 (48%) received NCBP therapy. Of the total patient population, 17 (13%) were admitted to the intensive care unit, 84 (63%) had a complicated UTI, and 64 (48%) had pyelonephritis. Baseline characteristics were similar between the two groups. There was no difference in clinical cure between the CBP and NCBP therapy groups (96% vs. 97%, p = 1.0). Additionally, no differences in secondary outcomes were observed. Subgroup analyses were performed in patients with specific pathogens, uncontrolled genitourinary source, complicated UTI, and pyelonephritis. These analyses did not reveal any differences in primary or secondary outcomes between the two groups.

**Conclusion:**

FEP and TZP may be reasonable CBP-sparing alternatives for the treatment of ESBL-E UTIs as clinical and microbiological outcomes were similar with these NCBP agents vs. CBPs in this study population.

**Disclosures:**

**Venugopalan Veena, PharmD**, **Melinta** (Other Financial or Material Support, Received a stipend for participation in a drug registry)**Merck** (Other Financial or Material Support, Received a stipend for participation in a drug registry)

